# Engineering Laccases: In Search for Novel Catalysts

**DOI:** 10.2174/138920211795564340

**Published:** 2011-04

**Authors:** Viviane Robert, Yasmina Mekmouche, Pierre R Pailley, Thierry Tron

**Affiliations:** Laboratoire Biosciences, Institut des Sciences Moléculaires de Marseille, Université Aix-Marseille, ISM2 CNRS UMR 6263, Marseille Cedex 20, France

**Keywords:** Biocatalysts, fungal laccases, heterologous expression, mutagenesis, multicopper enzyme.

## Abstract

Laccases (*p*-diphenol oxidase, EC 1.10.3.2) are blue multicopper oxidases that catalyze the reduction of dioxygen to water, with a concomitant oxidation of small organic substrates. Since the description at the end of the nineteenth century of a factor catalyzing the rapid hardening of the latex of the Japanese lacquer trees (*Rhus sp.*) exposed to air laccases from different origins (plants, fungi bacteria) have been continuously discovered and extensively studied. Nowadays, molecular evolution and other powerful protein modification techniques offer possibilities to develop tailored laccases for a wide array of applications including drug synthesis, biosensors or biofuel cells. Here, we give an overview on strategies and results of our laboratory in the design of new biocatalysts based on laccases.

Numerous detailed reviews about structure activity relationships, mechanism and applications of laccases are available in the literature [1-6]; therefore, laccases known properties will be only shortly described here. The enzyme active site comprises four copper atoms classified into types T1 (the blue copper), T2 or T3, according to their spectroscopic characteristics. Substrate oxidation (monoelectronic) occurs at the T1 copper site, while the T2/T3 tri-atomic cluster is responsible for the four electrons reduction of O_2_ [1]. The overall outcome of the catalytic cycle is a reduction of one molecule of dioxygen into two molecules of water, coupled with the oxidation of four substrate molecules (phenols or anilines) into four radicals that can form dimers, oligomers and polymers.

Hundreds of laccases encoding genes are now known and eleven crystal structures of laccases (one from bacterial and 10 from fungal origin) have been published so far. The scaffold of laccases consists in the repeat of a homologous domain (blue copper-binding domains D1 to D3) that shares distant homology to the single-domain cupredoxins [7, 8]. The evolutionary path from a single domain cupredoxin to a multi domains enzyme is thought to involve gene duplication, loss or acquisition of copper binding sites and domain recruitment [7, 8].

Laccases physiological roles encompass lignification, delignification, oxidative stress management, fungal morphogenesis and virulence [2]. Originally diverse, laccases are able to oxidize a disparate range of natural substrates: phenols, aromatic amines, metal ions. Functionally diverse, robust and environmentally friendly catalysts, laccases are largely studied for their potential uses in industrial processes [9] and increasingly studied as catalysts in organic synthesis applications [4]. Fungi are the source of predilection for laccases. The presence of laccases has been documented in virtually every fungus examined for it, including yeasts (e.g. *Cryptococcus*), molds (e.g. *Pennicillium*), mushrooms (e.g. *Agaricus*) and white rot fungi (e.g. *Pleurotus*) [10]. In general, fungal genomes code for several laccase isoenzymes. These enzymes are synthesized in variable quantities depending on the environmental conditions. For example, the genome of *Coprinopsis cinerea* contains 17 laccase coding genes [11]. Most of basidiomycetous laccases encoding sequences are under the control of promoters containing metal responsive elements (MRE) or xenobiotic responsive elements (XRE). It is therefore difficult to characterize or study the function of individual enzymes in such a context and often genes encoding for given isoenzymes are expressed in heterologous hosts.

Biotechnological applications (*e.g.* biosynthesis of a new dye [12]) require amounts of low cost laccases with properties like a high redox potential, optimal activity at neutral or alkaline pH, organic solvent tolerance and thermostability. These characteristics are unlikely to be all presents naturally in a single molecule and in search for the proper catalysts, optimization of nature derived scaffolds offers alternatives to natural biodiversity screenings. The work we conduct on laccases isoforms from the basidiomycete *Trametes* sp. C30 is an example on how one can find and express heterologously laccases genes, characterize and then use laccase enzymes to develop artificial catalysts for biotechnological applications.

## LACCASES FROM *TRAMETES* SP. C30

The white-rot basidiomycete C30 originates from a harvest of fungi found on evergreen oak (*Quercus ilex* L.) leaf litter in the Mediterranean area [13]. This particular isolate belongs to the *Coriolaceae *and more precisely to the *Trametes *genus [14]. *Trametes* sp. strain C30, produces several laccases isoenzymes (at least five, *vide infra*), the proportion of which depends on culture conditions [15, 16]. Thus, like in other basidiomycetes, a major isoenzyme is produced in basal cultivation conditions on malt medium (LAC1) [15] while the production of several other isoenzymes can be enhanced using inducers in the growth medium. For example, a second isoenzyme (LAC2) can be produced in purifiable amounts in cultures supplemented with both CuSO_4_ and *p*-hydroxybenzoate [16]. LAC1 and LAC2 are acidic proteins (4.2<pI< 4.7) with a spectroscopic signature (UV/visible and EPR) typical of multicopper oxidases [15, 17]. Considering the number of genes potentially transcribed from the genome of a basidiomycete (15 in *Coprinopsis cinerea*, [11]) and the number of molecules useable as inducers (virtually all aromatics), such a strategy to produce purifiable laccases is however limited (in our case to LAC1 and LAC2). In fact, as mentioned in the above introductory section, study of the properties of different laccase isoforms often rely on the cloning of laccases encoding genes, their further heterologous expression and the characterization of individual gene product (*vide infra*). The genome of *Trametes* sp. C30 is not sequenced, but 5 laccase encoding genes (*lac1*..., *lac5*) have been revealed by molecular analysis of the C30 genome and cloned [18]. *lac1*, *lac2 lac3, lac4* and *lac5* encode for typical laccase peptides with highly conserved regions where copper coordinating amino acids are clustered. Examination of the aminoacid sequences of the gene products reveals that the enzymes are 23% to 41% different from each other, LAC4 being the most divergent of the 5 isoenzymes (Table **1**).

LAC4 is also the less acidic isoenzyme with a theoretical isoelectric point (pI ≈ 5.6) 1.65 pH unit higher than that of LAC3 which is the most acidic isoenzyme (Table 2). With a wide range of pH at which they potentially have optimal activity, these isoenzymes constitute an interesting group to optimize for biotechnology applications.

### Heterologous Expression 

Laccase sequences from different fungi have been expressed in hosts like *Pichia pastoris* [19, 20] or filamentous fungi of the *Aspergillus* and *Trichoderma* genera [21, 22]. High yields of purified enzymes (up to nearly 1 gL^-1^ in *T. reseii* [22]) can be achieved in these latter systems. However, fundamental studies and optimization of the expression/kinetic parameters/stability in different conditions using various mutagenesis techniques (including directed evolution) are difficult to envisage in such producer strain. In fact, steps of mutant constructions and their selection are often performed in yeast. The specie is a matter of choice (*S. cerevisiae* [23], *Kluyveromyces lactis* [24], *Yarowia lipolytica* [25], *P. pastoris* [19]). Due in particular to the availability of a large set of molecular biology tools (replicative plasmid, inducible or constitutive promoter, easy transformation procedures…) *S. cerevisiae* is the simplest host successfully used to produce recombinant laccases from eukaryotes. On the other hand, the recombinant laccase produced in yeast is usually secreted directly into the culture broth, making easy both the screening for a desired activity (directly on plate or in multiwell microplates) and the subsequent purification steps. New enzymes or new functions can easily be created with a large set of mutagenesis techniques available (e.g. site directed or random mutagenesis, directed evolution, domain exchange) among which the natural homeologous recombination properties of *S. cerevisiae* that can be properly used to construct hybrid laccases [26]) are worth mentioning (*vide infra*).

Five genes encoding the *Trametes* sp. C30 laccase isoenzymes have been expressed in the common laboratory *S. cerevisiae* strain W303-1A [18]. A Petri dish plate inoculated with the recombinant yeasts transformed by pYES-DEST52 vector based constructions in which each of the five cDNAs *lac1, lac 2, lac 3, lac 4, lac 5* has been inserted under the control of the regulated *GAL1* promoter can be seen on Fig. (**1**). Variations in intensity of the brown coloration resulting from the oxidation of the substrate 2-methoxyphenol by laccases are linked to differences in production (rates) of active recombinant enzymes as well as to differences in their specific activity regarding the substrate (or a combination of the two factors). This example underlines how advantageous it is to start heterologous expression of laccases with a set of genes presenting a diversity in sequence. From this experiment, the cDNA encoding LAC3 has appeared an obvious choice as a reference sequence for further developments of artificial laccases.

Ability to efficiently produce laccases in a heterologous host largely depends on the nature of changes introduced in the original DNA or aminoacid sequence. Improving laccase secretion in heterologous hosts has been obtained replacing the DNA encoding the fungal signal sequence by that encoding for a highly secreted protein of the host [27, 18, 23]. Further improvement in secretion can be obtained during molecular evolution of the chimeric sequence [28]. Differences in expression of recombinant enzyme coding sequences are also likely often related to inappropriate codon usage. Low-frequency codons can cause translation pauses depending on their position and abundance. As a matter of fact, synonymous mutations to more frequently used codons have been found to improve production of a recombinant laccase up to eightfold during the functional expression of a *M. thermoplila* laccase gene in *S. cerevisiae* modulated by directed evolution [29]. More affordable than ever, synthetic genes encoding laccase sequences with an optimized codon set are certainly a good starting point for expression optimization.

Yeast expression systems, and in particular *S. cerevisiae* and *P. pastoris*, are known to hyperglycosylate recombinant foreign proteins. As recently pointed out in a review on designer laccases [6], this phenomenon that hasn’t received much attention so far shouldn’t be overlooked since carbohydrate moieties might interfere with protein secretion, protein folding and function including that to be ultimately acquired by tailoring. The use of drugs blocking the glycosylation process (like tunicamycin) or the design of glycosylation deficient variants (in particular by mutagenesis at N-glycosylation sites) may be of great interest in the study of glycosylation in some instances. In other instances, the simple characterization of purified glycovariants may be informative. Thus, the comparison of differently glycosylated forms of the same LAC3 mutant enzyme recently obtained in our lab in substantial amounts suggests that overglycosylation does not affect much enzyme’s properties but rather its production^1^.

Ultimately, characterized laccases variants obtained in yeasts could be mass produced in filamentous fungi. We recently produced and purified ourselves the *Trametes* sp. C30 LAC3 from the host *A. niger* with a yield two order of magnitude higher than that obtained in *S. cerevisiae*^2^.

## FROM LACCASE TO ARTIFICIAL ENZYMES

Cumulated examples of laccase modifications found in the literature constitute precious information for anyone who wants to start customizing or evolving the enzyme. Mutagenesis of laccase has started in the 90’s and has been used so far both for structure-activity relationships studies on the enzyme and for tuning their properties in search for improved catalysts. Rationally or randomly generated mutations have been thus used to probe the first and second coordination spheres of the Cu T1 [30, 31] or shape substrates interacting areas at the surface of the enzyme [32, 33]. In some instances, these techniques have also permitted to directly optimize enzyme kinetic parameters [29, 32] or to increase laccase resistance to organic solvents [34]. The plasticity of the enzyme has been also challenged by drastic modifications like deletions or additions. From deletion studies, it appears that the integrity of the C-terminus of laccases is important to the activity of the *Melanocarpus albomyces* laccase [35], the stability of the *Pleurotus ostreatus* laccase POXA1b enzyme at alkaline pH [36] and for maintaining a high Cu T1 midpoint potential (E°) in the LCCIa from *Trametes versicolor* [37]. On the other hand, fusing the C-terminus of a laccase to binding peptides have been used to target an active bleaching agent (laccase) to cellulosic materials [38] or to stained fabrics [39].

Offering a nice angle of view on the plasticity of the enzyme, the evolutionary path of laccase within the large Blue Copper Binding Domain protein family is a great source of inspiration for transforming laccases into artificial catalysts with new properties. Evolution of a single domain cupredoxin (CUP, the most ancient fold of the BCBD) into multiple CUP domain enzymes (laccases, nitrite reductase, cerulopasmin) has involved gene duplication, loss or acquisition of copper binding sites and domain recruitment [7, 8]. Eventually, the function depends on the number of CUP domains, the number and type of copper atoms, the fusion to non CUP protein domain (e. g. the human coagulation factor VIII) and on the interaction with an auxiliary (e. g. plastocyanin and cytochromes).

The five genes encoding *Trametes* sp. C30 laccases represent an interesting natural diversity we intend to use to create laccase-based catalysts. In a first approach, we used basic protein engineering techniques -like fusion, deletion, domains swapping- to explore properties of simple artificial laccases expressed in heterologous hosts. Illustrative examples are following.

### Tagging Laccase

Controlling the molecular structure, organization, and orientation of laccases on a solid surface without hampering their catalytic properties is a key issue in the development of biomolecular electronics or biofuel cells. Initially developed for the purification of genetically engineered proteins, the formation of ternary metal–chelate complexes between nitrilotriacetic acid (NTA) and histidine-tagged recombinant proteins is an efficient strategy for controlling protein orientation at interfaces (e.g. commercially used on chips for Surface Plasmon Resonance). We have been using self-assembled monolayers (SAM) of NTA-terminated alkanethiols on a gold surface for site-specific immobilization of recombinant histidine-tagged laccase enzyme (Fig. **2**).

Using this technology it is possible to form active monolayers of N or C terminally 6 His tagged laccase oriented at the surface the modified electrode [40]. The catalytic efficiency of the C or N tagged enzyme in solution is 0.5 to 1 time that of the untagged enzyme^1^ and is apparently not affected by the immobilization procedure [40]. However, electron transfer from the electrode had to be mediated by the redox mediator [Os^III^ (bpy)2pyCl]^2+^ for both C and N tagged enzymes. As this is likely related either to the length of the spacer or to an inappropriate location of the tag regarding the T1 Cu binding site (*i. e.* too far away), these two parameters can be the targets for tuning in order to get an efficient Direct Electron Transfer (DET). Generally speaking, it appears that the nature of the tag (peptide or protein domain) or its length (so far up to 78 residues [38]) are two important variables in the design of functional hybrids. As an illustration, it is worth mentioning here that a LAC3-GST (plus 26kDa) hybrid is only transiently detected in the supernatant of a *S. cerevisiae* grown at 20°C and is unstable during its purification on affinity column [41].

### Shuffling Domains

*In vivo* recombination strategies offer interesting tool to construct original hybrid variants. In the model organism *S. cerevisiae*, homeologous recombination allow the yeast to recombine similar but not identical DNA with rates proportional to the length of homology [42, 43]. Usually, *in vivo* generated chimera results from the shuffling of large blocks of sequence corresponding to one or more structural domain (Fig. **3A**). We have constructed laccase chimeras through yeast mediated homeologous recombination of *Trametes* sp. strain C30 laccase cDNAs sharing 65–71% identity [26] (Fig. **3A**, **B**). Hybrid laccase coding genes *lac131*, *lac232* and *lac535* obtained by recombination all contain a *clac3* central sequence (700 to 800 bp long, about half of the total length) with junctions found to map within short stretches of identity varying from 5 bp to 45 bp.

**Fig. (1).**
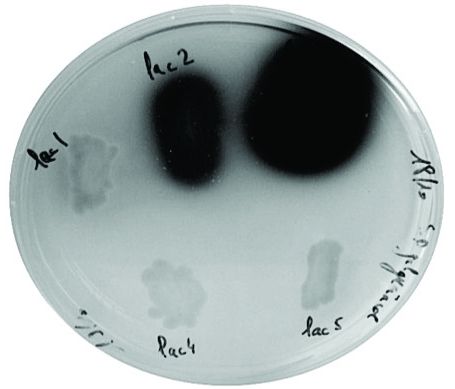
recombinant yeasts expressing *Trametes* sp. C30 lacase genes grown on selective medium (SGal URA). From top to bottom and left to right: *URA3* based pYES-DEST52 constructs containing each of the five cDNAs *lac1, lac 2, lac 3, lac 4, lac 5* inserted under the control of the regulated *GAL1* promoter. The halo around colonies results from the oxidation of the 2-methoxyphenol of the medium by secreted active laccases.

**Fig. (2).**
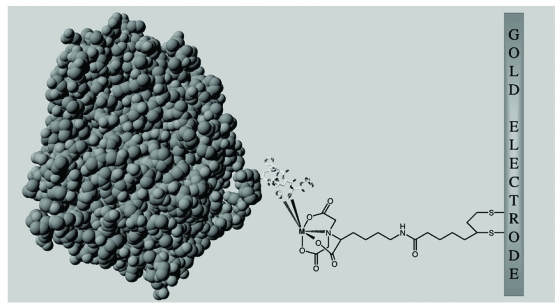
immobilization of a HIS-tagged laccase at the surface of a gold electrode. The enzyme 3D model is depicted in gray (space fill-in); the 6 histidine residues of the tag are represented as sticks; the extended chemical structure of the NTA alkane-thiol spacer is represented in black.

**Fig. (3).**
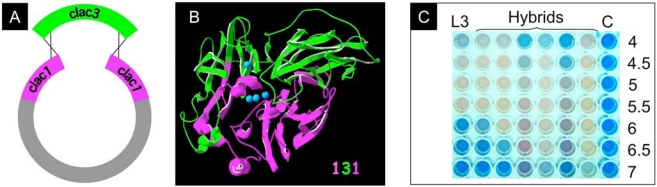
homeologous recombination in yeast. **A**: scheme of double crossing over required for plasmid repair at homeologous sites. **B**: model of the hybrid LAC131; the amino and carboxy-terminal parts corresponding to the LAC1 sequence is depicted in purple, the central part corresponding to the LAC3 sequence is depicted in green, Cu atoms are depicted in cyan. **C**: extended activity of hybrids at non permissive pH (biotransformation of AB64 (blue anthraquinonic dye) into LAR1 (red azo dye) see reference 12) : L3= LAC3 (reference); Hybrids= chimeric laccases obtained by homeologous recombination, [26]; C=control without laccase ; numbers on the right refer to pH values).

Crossovers leading to functional hybrids occur at positions that minimize disruption of interactions. In the chimera, the integrity of domain D1 and that of the very end part of domain D3, two regions that are interacting in the natural laccase fold, are preserved (Fig. **3B**). Exchange of structural blocks allows to create hybrid sequences that are better expressed in the host than parental sequences (see Fig. **1** for an estimation of the LAC1, LAC2 or LAC5 parental enzymes production) [26]. Hybrid enzymes have properties distinct from those of the parental enzymes (*i. e.* not predictable from the parental enzyme’s properties), like for example an enhancement of the enzyme stability at neutral or alkaline pH (Fig. **3C**, see also ref. [26]). Thus, the catalytic efficiency of the best performing hybrid (LAC131) represent more than 12 times that of the parental enzyme (LAC3). When compared to studies involving mutagenesis, such a factor is one of the highest ever observed in a single step.

**Fig. (4).**
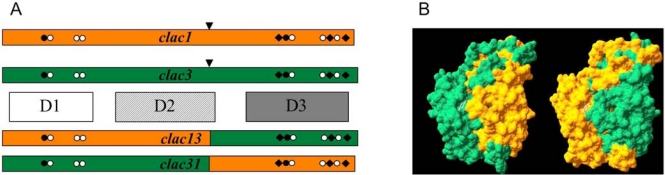
hybrids made *in vitro.* A: scheme of the restriction/ligation construction; ▼, position of the Bam*HI* restriction site. B: models of the hybrids LAC13 and LAC31.

Large blocks of sequence can be also shuffled *in vitro*. Although 32% different in sequence, the *clac1* and *clac3* cDNAs are cut by the restriction enzyme Bam*HI* at the same location. Cross ligations of the fragments allow the creation of the chimeric genes *clac13* and *clac31* encoding for the hybrids LAC13 and LAC31 (Fig. **4**). If LAC31 can be produced as an active enzyme with properties similar to LAC3, the LAC13 hybrid is apparently either totally inactive or non produced^3^. A negative result is always difficult to interpret. However, the recent publication of strikingly identical observations made on two sequences encoding *Lentinula edodes* laccases shuffled over the same Bam*HI* crosspoint to give only an active LAC41 on the two possible hybrids retain the attention [44]. Indeed, this genera independent fate suggests the presence in these sequences (both in *L. edodes* and in *Trametes* sp. C30) of a structural element that can prevent the expression of an active enzyme. Whatever the nature of the provoked impairment could be, its gives new prospects in the study of the synthesis of a multicopper oxidase.

### Trimming the C-Terminus

Unlike ascomycetes, a processing of the C-terminal sequence is apparently not mandatory to the activity of basidiomycetous enzymes. Deleting a chunk of the LAC3 C-terminal sequence does not hinder production of the enzyme but, to the contrary, apparently enhance it^1^. The C-terminus of the mature protein is nonetheless sensitive to modifications. A deletion of the last 13 residues makes the cysteine involved in a disulfide bond connecting domain 3 to domain 1 the ultimate residue of the polypeptide. Although a disulfide bridge might not be mandatory to the activity in laccase, this structural element has been arbitrarily chosen as a physical limit here since, spatially close both to the entrance of one of the channel leading to the trinuclear cluster and to the T1 copper, the cysteine is potentially a good anchor point for further modifications [37]. On the other hand, trimming 13 aminoacids of the LAC3 C-terminus results in a drop of catalytic efficiency (*i. e.* *k_cat_/K_M_*) 0.3 to 1 order of magnitude depending on substrate and affect the pH and the thermal dependent stability of the enzyme^1^. Technically, the deleted LAC3 is nearly identical to the POXA1b  16 from *Pleurotus ostreatus* [36] and to the LCCIa from *Trametes versicolor* [37] which similarly are affected in their catalytic activity. For LCCIa, the modification of the C terminus has been associated to modification of the electrochemical properties of the enzyme with a drop of nearly 300mV of its midpoint potential [37]. Interestingly, unlike LCCIa, the 13 residues deleted LAC3 is still able to oxidize ABTS (E°ABTS= 0.68V *vs* NHE) and even syringaldazine (E°SGZ= 0.7V *vs* NHE) suggesting that the LAC3 midpoint potential (*i. e.* the T1 copper E°) is probably not that diminished by the deletion^1^. It seems that the C-terminus of laccase is fairly sensitive to modifications and therefore one must be particularly cautious when planning any engineering of this site.

## PROSPECTS

Any alteration introduced into a wild type protein can, in ways that often cannot be predicted, disrupt the fine balance that nature has achieved. As a response to difficulties encountered using strict rational design approaches, there is an increasing trend towards the use of molecular biology strategies that mimic evolutionary processes. Directed evolution is an effective strategy to engineer and optimize protein properties [45]. Such strategies are particularly successful in achieving improvements in thermostability, altering substrate specificity and improving activity in organic solvents. These techniques are also often employed in *S. cerevisiae* to improve expression of poorly expressed laccase.

Improved laccases have a recognized industrial potential but their development essentially concern bioremediation or oxidative degradation processes. Despite the fact that fungal laccases have been found to catalyze oxidation of aromatic alcohol to aldehydes, synthesis of substituted imidazoles, dimerisation of penicillin or cephalosporin monomers etc., they are largely not used as synthetic tools. This is mainly due to the fact that the phenoxy radical produced by the enzyme is persistent, of difusible nature and it can undergo C-C or C-O coupling in absence of any selectivity control by the enzyme. Regio- and stereoselective phenol coupling is however observed in plant secondary metabolism [46, 47) but also in bacteria, lichen, and fungi [48]. Therefore, it would be of a particular interest to succeed in introducing such selective properties into laccase. A combination of phenol coupling factors like for example the plant dirigent proteins [46, 47] and laccases into a single selective catalyst would undoubtedly represent a major breakthrough in biocatalysis. Information on the selectivity of the coupling mechanism is presently limited [49] but there is no doubt that structure-function relationships studies on these radical binding factors will in the near future help to develop strategies to mimic this function in laccases. This prefigure an entirely new way to look into this industrial enzyme in which laccases *per se *are not improved any longer but instead are used as a powerful electrochemical engine coupled to modulators to create laccase based biocatalysts with an entirely new set of functions. In this direction, improved versatile protein chemical modifications offer multiple possibilities to graft laccase with functionalizing objects like transition metal complexes or synthetic receptors to enlarge the scope of the chemical reactions catalyzed.

## Figures and Tables

**Table 1.** *Trametes* sp. C30 Laccase Sequences Identities (%). Similarities are in italic

	LAC1	LAC2	LAC3	LAC4	LAC5
**LAC1**		70	70	63	75
**LAC2**	*80*		77	59	65
**LAC3**	*81*	*85*		59	68
**LAC4**	*74*	*69*	*70*		62
**LAC5**	*87*	*77*	*78*	*73*	

**Table 2.** *Trametes* sp. C30 Laccases Properties

	Length (aa)	Calc. Mw (Da)	Estimated pI
**LAC1**	496	53189	4.70
**LAC2**	505	54588	4.26
**LAC3**	501	53767	3.94
**LAC4**	494	53983	5.59
**LAC5**	497	53477	4.72

## Footnotes

1 Liu, Y.; Cusano, A. M.; Wallace, E.; Mekmouche, Y.; Robert, V.; Tron, T.; unpublished articles.

2 Mekmouche, Y.; Zhou, S.; Record, E.; Tron, T.; unpublished articles.

3 Mouric, S.; Tron, T., unpublished results.
